# Effect of 6% hydroxyethyl starch 130/0.4 in 0.9% sodium chloride (Voluven®) on complications after subarachnoid hemorrhage: a retrospective analysis

**DOI:** 10.1186/2193-1801-2-314

**Published:** 2013-07-15

**Authors:** Shariq A Khan, Owoicho Adogwa, Tong J Gan, Ulysses T Null, Terence Verla, Sankalp Gokhale, William D White, Gavin W Britz, Ali R Zomorodi, Michael L James, David L McDonagh

**Affiliations:** Department of Anesthesiology, Duke University Medical Centre, Durham, NC USA; Department of Anesthesiology, Singapore General Hospital, Singapore, Singapore; Departments of Surgery (Neurosurgery) & Radiology, Duke University Medical Centre, Durham, NC USA; Duke University Medical School, Durham, NC USA; Division of Neurocritical care, Department of Neurology, Duke University Medical Center, Durham, NC USA

**Keywords:** Subarachnoid hemorrhage, Hydroxyethyl starch, 6% HES 130/0.4, Voluven, Fluid therapy, Delayed cerebral ischemia, Hydrocephalus, Rebleeding, Mortality

## Abstract

**Background:**

6% Hydroxyethyl Starch 130/0.4 in 0.9% Sodium Chloride (Voluven®; 6% HES 130/0.4) is a colloid often used for fluid resuscitation in patients with subarachnoid hemorrhage (SAH), despite a lack of safety data for this use. The purpose of our study was to evaluate the effect of 6% HES 130/0.4 on major complications associated with SAH.

**Methods:**

Medical records of all patients presenting between May 2010 and September 2012 with aneurysmal SAH were analyzed. Patients were divided in two groups based on the administration of 6% HES 130/0.4; HES group (n=57) and Non-HES group (n=72). The primary outcome included a composite of three major complications associated with SAH: Delayed Cerebral Ischemia (DCI), Hydrocephalus (HCP) requiring cerebrospinal fluid (CSF) shunting, and Rebleeding.

**Results:**

The study groups were similar with respect to most characteristics except the incidences of hypertension, ischemic heart disease, Fisher grade and lowest hemoglobin during stay. The odds of developing the primary composite outcome was higher in the HES group [OR= 3.1(1.30-7.36), p=0.01]. The patients in the HES group had a significantly longer median duration of hospital (19 vs 14 days) and Neurointensive Care Unit stay (14 vs 10 days) compared to the Non HES group.

**Conclusion:**

We observed increased complications after SAH with 6% HES 130/0.4 (Voluven®) administration. An adequately powered prospective randomized controlled trial into the safety of 6% HES 130/0.4 in this patient population is warranted.

**Electronic supplementary material:**

The online version of this article (doi:10.1186/2193-1801-2-314) contains supplementary material, which is available to authorized users.

## Background

Aneurysmal Subarachnoid hemorrhage (SAH) has an incidence of nine cases per 100,000 patient years (de Rooij et al. 2007), with 65% survival after the initial hemorrhage (Lovelock et al. 2010; Nieuwkamp et al. 2009). Most fatality occurs within 2 weeks after ictus, with 10% occurring before receiving medical care and 25% within 24 hours of the event (Broderick et al. 1994). Initial management after resuscitation and stabilization is the prevention of re-bleeding, prevention and management of vasospasm, and the treatment of other complications like hydrocephalus (HCP) (Solenski et al. 1995; van Gijn et al. 1985).

Maintenance of euvolemia remains a mainstay of treatment of these patients during their initial stay (Storrow and Wrenn 2006). Clinicians have used several different fluid regimens and formulations to achieve this goal. Hydroxyethyl Starch (HES) based resuscitation fluids have been available for several years, with subsequent generations of HES differing in their mean molecular weight (Mw), Molar substitution (MS), C2/C6 hydroxyethylation ratio and solvent (saline or balanced salt solution). The current generation HES are more rapidly degradable with a lower Mw (130 kDa) and a lower MS (<0.5). Voluven® (Fresenius Kabi, Bad Homburg, Germany) is a colloid solution which consists of a maize derived 6% HES (Mw: 130 kDa, MS: 0.40, C2/C6 ratio 9:1) suspended in normal saline, which has been available in the United States since 2007. Despite its contraindication for use in patients with intracranial hemorrhage (Anonymous 2013), it is used fairly commonly in many neuro-intensive care units including our own once the aneurysm has been secured. Moreover, recently there have been a number of publications in literature which describe the use of 6% HES 130/0.4, in patients which have had SAH (Lehmann et al. 2013; Anonymous 2012; Moretti and Pizzi 2010).

Recent publications with regards to the use of HES in the critically ill patients have indicated a tendency toward harm (Perner et al. 2012; Myburgh et al. 2012). To the best of our knowledge there are currently no safety data with regards to the use of 6% HES 130/0.4 in patients with SAH. We therefore conducted a retrospective observational study to assess the impact of 6% HES 130/0.4 administration on the incidence of complications in patients admitted for aneurysmal subarachnoid hemorrhage. Our a priori defined primary outcome was a composite of serious complications associated with SAH (Delayed Cerebral Ischemia (DCI), Rebleeding, and HCP requiring CSF shunting).

## Methods

After obtaining Institutional Review Board approval, all patients who had SAH at Duke University Hospital between May 2010 (when we implemented use of 6% HES 130/0.4) and September 2012 were identified using the appropriate International Classification of Diseases (ICD 9) code. The list of patients was further checked manually by the authors using electronic records (eBrowser, version 7.07, Pegasus Imaging Corporation, Tampa, FL, USA). Patients were excluded from analysis if they were incorrectly coded by ICD 9 classification, had SAH secondary to trauma or anticoagulation, if they had an SAH that was non-aneurysmal in nature, or where the source of SAH was not apparent. Baseline hospital admission characteristics including preexisting medical problems, home medication use, SAH severity, and admission laboratory values were obtained for each eligible patient. This information was obtained by manual searching of electronic medical records and by an automated search of Duke University hospital’s Decision Support Repository (DSR) using the Duke Enterprise Data Unified Content Explorer (DEDUCE) portal. The DSR is a quality assured custom-built data warehouse containing integrated clinical and financial data of all patients admitted to the Duke health care system (Horvath et al. 2011). Individual values which deviated noticeably from the normal or any missing data points were further cross checked manually by a separate investigator after completion of the preliminary data collection. Medical conditions and medication used at the time of admission were considered to be present if they were recorded in the electronic medical records.

### Clinical management

All patients with a presumptive diagnosis of SAH presenting to our institution are admitted to the Neurosciences Intensive Care Unit (NICU) after undergoing confirmatory radiological investigations. All patients receive a normal saline infusion supplemented with colloids including 5% Human Plasma Protein Fraction (Plasmanate®, Talecris Biotherapeutics, Research Triangle Park, NC, USA) or 6% HES 130/0.4 (Voluven®) to maintain a positive daily fluid balance and adequate urine output. All patients undergo daily transcranial Doppler ultrasound and routine laboratory investigations (blood counts and electrolytes) for 14 days. Blood pressure is maintained below a systolic blood pressure (SBP) of 140 mmHg until the aneurysm is secured (using appropriate medications if necessary), following which the blood pressure targets are relaxed to a SBP < 160 mmHg. Patients undergo frequent neurological assessments during their stay; any deterioration in the exam is aggressively investigated with appropriate tests (CT angiography and/or conventional angiography). Hypertensive euvolemic therapy is initiated in response to suspected cerebral ischemia due to vasospasm. Refractory symptomatic vasospasm is treated with intra-arterial vasodilators and/or angioplasty. External ventricular drains (EVD) are placed to treat HCP. Patients unable to tolerate drain weaning and removal are given ventriculoperitoneal shunts. Intracranial hypertension is treated with repeated boluses of 20% mannitol (0.25–1.5 g/kg) or 23.4% hypertonic saline targeted to serum osmolality of < 330 mOsm/L. Red blood cell transfusion is targeted to a hemoglobin to >8 g/dl (>9 g/dl in patients with significant heart disease).

We studied the effect of 6% HES 130/0.4 administration on complication rates in patients with aneurysmal SAH. Patients were classified into the 6% HES 130/0.4 group (HES) if they received any amount of 6% HES 130/0.4 during their hospital stay for SAH, while all others were classified into the Non-HES group.

The primary outcome was a priori defined composite of three major complications (Delayed Cerebral Ischemia, Rebleeding, & HCP requiring permanent CSF shunting) (Defined in Appendix 1). We also compared Modified Rankin scores on discharge, blood product utilization, change in serum creatinine (∆Cr), 30 day mortality and durations of hospital and ICU stay between the two groups.

### Statistical analysis

Data are presented as median [interquartile range (IQR)] or mean (standard deviation) for continuous variables and as count (percentage) for categorical variables. Mann–Whitney U test, Fisher exact or Chi-square tests were used for intergroup comparisons as appropriate. Co-variables that were significantly different between the two study groups were tested in a multivariate logistic regression model with HES use, and non-significant variables were sequentially removed. Interactions between HES use and the significant variables were also tested. Statistical significance was defined as a P<0.05. All analysis was done using SAS statistical software version 9.3 (SAS Institute, Cary, NC).

## Results

There were 544 cases with an ICD 9 diagnosis code of SAH identified from the database during the study period. After removal of cases meeting the exclusion criteria, 129 cases of SAH in 129 patients were included in the final analysis. 57 patients (44.2%; HES group) had 6% HES 130/0.4 administered during their hospital stay while 72 patients (55.8%; Non-HES group) did not. Baseline demographics, medical conditions, home medication, and laboratory values on admission are presented in Table 1.

**Table 1:** **Demographics and laboratory values between the study groups**

	Non-HES group (n=72)	HES group (n=57)	p value
*Demographic characteristics*			
Age (years)	57 (48–63)	57 (46–65)	0.58
Weight (kilogram)	75 (62.5-87)	79 (63.5-97.5)	0.18
Male	24 (33.3%)	18 (31.6%)	0.83
Female	48 (66.4%)	39 (68.4%)	
Caucasian	38 (52.8%)	32 (56.1%)	0.88
African-American	27 (37.5%)	19 (33.3%)	
Others	7 (9.7%)	6 (10.5%)	
*Pre-existing medical conditions at admission*			
No past medical history	7 (9.7%)	3 (5.3%)	0.34
Ischemic heart disease	4 (5.6%)	10 (17.5%)	0.03
Congestive heart failure	4 (5.6%)	4 (7%)	0.73
Cerebro-vascular accident- stroke	2 (2.8%)	2 (3.5%)	0.81
Cerebro-vascular accident- transient ischemic attack	0 (0%)	2 (3.5%)	0.11
Previous history of Subarachnoid Hemorrhage	0 (0%)	0 (0%)	NA
Epilepsy	1 (1.4%)	1 (1.8%)	0.86
Hypertension	40 (55.6%)	43 (75.4%)	0.02
Chronic renal failure- no dialysis	2 (2.8%)	3 (5.3%)	0.46
Chronic renal failure- Peritoneal or Hemodialysis	0 (0%)	0 (0%)	NA
Diabetes- not on insulin	2 (2.8%)	3 (5.3%)	0.47
Diabetes- treated with insulin	3 (4.2%)	5 (8.8%)	0.28
COPD ( no home oxygen)	3 (4.2%)	4 (7%)	0.48
COPD (home oxygen)	0 (0%)	0 (0%)	NA
Smoker	22 (30.6%)	11 (19.3%)	0.14
Family history of Subarachnoid Hemorrhage	6 (8.3%)	2 (3.5%)	0.26
*Home medication at admission*			
Patient not on any home medications	18 (25%)	10 (17.5%)	0.31
Aspirin	17 (23.6%)	20 (35%)	0.15
Clopidogrel	0 (0%)	2 (3.5%)	0.11
Statin	22 (30.6%)	12 (21%)	0.22
*Subarachnoid Hemorrhage grading at admission*			
*Hunt-Hess grade*			
Hunt-Hess grade 1	6 (8.3%)	4 (7%)	
Hunt-Hess grade 2	36 (50%)	32 (56.1%)	
Hunt-Hess grade 3	17 (23.6%)	13 (22.8%)	
Hunt-Hess grade 4	6 (8.3%)	2 (3.5%)	
Hunt-Hess grade 5	7 (9.7%)	6 (10.5%)	0.82
*Fisher grade*			
Fisher grade 1	4 (5.5%)	1 (1.8%)	0.04
Fisher grade 2	12 (16.7%)	2 (3.5%)	
Fisher grade 3	45 (62.5%)	47 (82.4%)	
Fisher grade 4	11 (15.3%)	7 (12.3%)	
*Treatment of aneurysm during this hospital stay*			
Open Craniotomy and aneurysm clipping	31 (43%)	32 (56.1%)	0.14
Endovascular coil embolization	35 (48.6%)	22 (38.5%)	0.25
*Laboratory investigation during hospital stay*			
Hemoglobin at admission (g.dl^-1^)	13 (11.6-14)	12.7 (11.5-14.3)	0.84
Lowest Hemoglobin during hospital stay (g.dl^-1^)	9.5 (8.3-10.7)	8.3 (7.6-9.4)	0.001
Platelet count at admission (× 10^9^/L)	243 (206–283)	235 (194–272)	0.59
Prothrombin Time at admission (sec)	11.9 (11.1-12.8)	12.2 (11.6-13)	0.16
Partial Thromboplastin Time at admission (sec)	26.8 (24–28.8)	25.7 (23.5-27.6)	0.26
Serum Creatinine at admission (mg.dl^-1^)	0.8(0.6-1)	0.8(0.7-0.9)	0.46
Highest Serum Creatinine during hospital stay (mg.dl^-1^)	0.9 (0.8-1.2)	0.9 (0.75-1.1)	0.70

Median (Interquartile Range) was reported for continuous variables and count (%) for categorical variables. Mann–Whitney U test was used to compare continuous variables and chi-square test for categorical variables.

*COPD* Chronic obstructive pulmonary disease.

*ARB/ACEI* Angiotensin receptor blocker/Angiotensin converting enzyme inhibitor.

*NA* Not applicable.

In our study population SAH occurred more commonly in females (67.4% vs. 32.6% males) with an average age of 56.3 yr (SD ±13). Racially, aneurysmal SAH was most frequent in Caucasian patients compared to African American and other groups (54.3%, 35.7% and 10%, respectively). Aneurysms were located (in decreasing order of frequency) in the posterior communicating artery (35.7%), anterior communicating artery (32.5%), middle cerebral artery (17%), basilar artery (10.9%) and other sites (3.9%). Overall 30-day mortality was 13.9%. The overall median durations of hospital and NICU stays were found to be 15 days (IQR: 13 to 23) and 12 days (IQR: 8.50 to 16), respectively. The aneurysm was secured in 63 patients by craniotomy with clip ligation and in 57 patients by endovascular coiling. In 9 patients (3 in the HES group, 6 in the Non HES group) the aneurysm was not treated.

**Figure 1:**
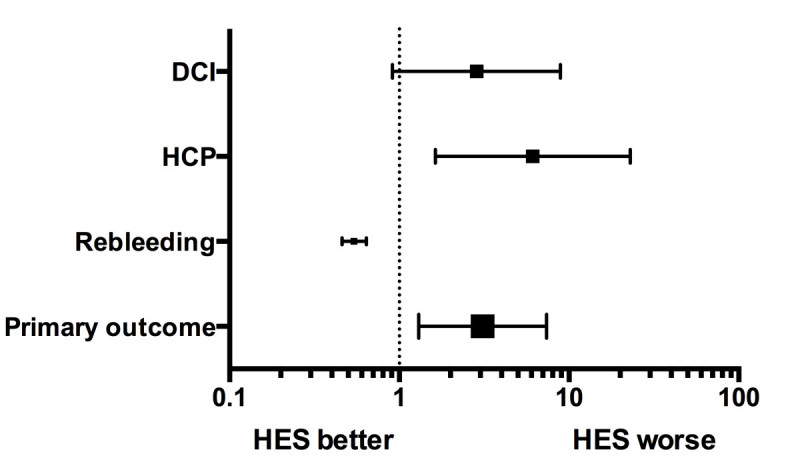
**Forest plot illustrating unadjusted odds ratio of the primary outcome, rebleeding, delayed cerebral ischemia (DCI), and HCP requiring permanent CSF shunting in the study population.**

A comparison of outcomes of interest between the study groups is described in Tables 2 & 3. The composite primary outcome occurred in 19 (33.3%) and 10(13.8%) patients in the HES and non-HES groups, respectively (p=0.01). The odds of the primary composite outcome were more in the HES group [OR=3.1(95CI: 1.30-7.36), p=0.01] Figure 1. The Median amount of 6% HES 130/0.4 administered to the HES group was 1250 ml (IQR: 500–2500).The median 6% HES 130/0.4 doses in the patients in the HES group and in those which suffered the primary outcome were 15.63 ml/kg (IQR: 6.62-26.60) and 16.26 ml/kg (IQR: 8.33-27.16), respectively. We found that the nadir hemoglobin level was significantly lower in the HES compared to the Non HES group during the hospital stay [8.3 (IQR: 7.6 to 9.4) and 9.5 (IQR: 8.3 to 10.7), respectively].

**Table 2:** **Clinical outcomes between study groups**

	Non HES group (n=72)	HES group (n=57)	Odds ratio (95%CI)	p value
Primary outcome	10 (13.8%)	19 (33.3%)	3.1 (1.30-7.36)	0.01
Rebleeding	4 (5.6%)	0 (0%)	0.54 (0.46-0.64)	0.13
HCP requiring CSF shunting	3 (4.2%)	12 (21%)	6.1 (1.63-22.95)	0.004
Delayed cerebral ischemia	5 (6.9%)	10 (17.5%)	2.86 (0.91-8.88)	0.09

Data reported as count (%).

*HCP* Hydrocephalous.

*CSF* Cerebrospinal fluid.

*CI* Confidence interval.

**Table 3:** **Length of hospital stay, percent creatinine rise, modified Rankin score at discharge and blood product utilization between the study groups**

	Non-HES group (n=72)	HES group (n=57)	*p value
Length of ICU stay^†^	10 (8–13)	14 (11–19.5)	0.001
Length of hospital stay^†^	14 (12–17)	19 (14–26)	0.001
30 day mortality	11 (15.3%)	7 (12.2%)	0.79
Percentage ∆Cr in patients with primary outcome^†^	7.1 (0–27)	12.5 (0–37.5)	0.74
Modified Rankin score (discharge)			
0	8 (11.1%)	2 (3.5%)	0.38
1	26 (36.1%)	30 (52.6%)	
2	14 (19.4%)	7 (12.3%)	
3	7 (9.7%)	4 (7%)	
4	5 (6.9%)	6 (10.5%)	
5	1 (1.4%)	1 (1.7%)	
death	11 (15.3%)	7 (12.3%)	
Blood product usage^‡^			
5% PPF	24.31 (119.85)	26.32 (102.25)	0.51
PRBC	184.65 (428.64)	388.12 (610.98)	0.006
FFP	30.67 (131.42)	82.84 (299.60)	0.43
Platelet	34.04 (105.06)	109.30 (445.32)	0.57

*Mann–Whitney U test was used to compare continuous variables and chi-square test for categorical variables. Data reported as †Median (Interquartile range) ‡Mean (Standard deviation) for continuous variables and count (%) for categorical variables.

*ICU* Intensive care unit.

*∆Cr* Creatinine rise.

*PPF* Plasma protein fraction (Human).

*PRBC* Packed red blood cells.

*FFP* Fresh frozen plasma.

Patients in the HES group received significantly more red blood cell transfusion that the Non HES group. There was no statistically significant difference in the amount of 5% Plasma Protein Fraction (Human), platelets, or fresh frozen plasma between the study groups (Table 3). Median creatinine rise (∆Cr) in patients which had the primary outcome in the HES and Non HES groups were 12.5% (IQR: 0–37.5) & 7.1% (IQR: 0–27), p=0.74, respectively.

### Multivariate analysis

**Figure 2:**
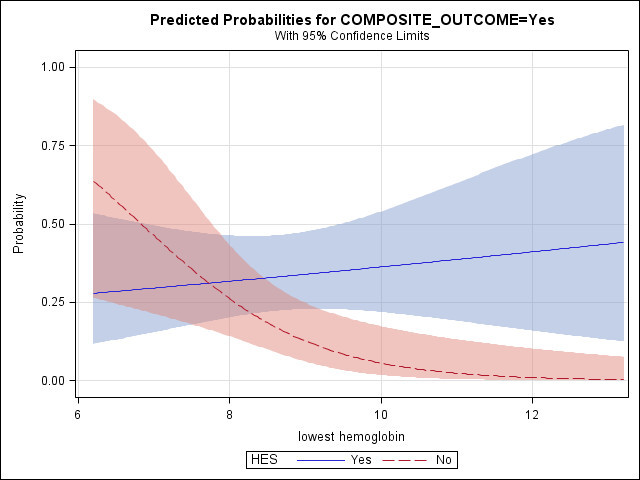
**Probability of primary composite outcome with HES use at different levels of lowest hemoglobin.**

Covariates tested with HES use in the multivariable logistic model included lowest hemoglobin during stay (LHB), Fisher Grade, Ischemic heart disease, and Hypertension. Of these, only LHB showed a significant association with the outcome in an interaction with HES (interaction p=0.0085). The model predicted an increased risk of the primary outcome with HES use at higher levels of LHB (see Figure 2). Estimated odds ratios of primary outcome with HES use were 3.55 (95CI: 1.32- 9.60) at LHB=9 gdl^-1^, compared to 5.84 (95CI: 1.78-19.1) at LHB=9.5 gdl^-1^. The model c-index (ROC area) was 0.729.

## Discussion

We found that there was a statistically significant increased incidence of the primary outcome in patients who received HES. Patients in the HES group also had significantly longer durations of hospital and NICU stay compared to the Non-HES group (Table 3). However, there was no statistically significant difference in the overall 30-day mortality between the study groups (Table 3).

Voluven® (Fresenius Kabi, Bad Homburg, Germany), a colloid solution consisting of a maize derived 6% HES (Mw: 130 kDa, MS: 0.40, C2/C6 ratio 9:1) suspended in normal saline, has been available in the United States since 2007. Similar to the earlier HES based fluids, it has a longer intravascular half-life (compared to isotonic crystalloids) and is therefore considered a suitable resuscitation fluid in hypovolumic states (Guidet et al. 2012). 6% HES 130/0.4 is not recommended for use in patients with intracranial hemorrhage due to its possible effects on coagulation. 6% HES 130/0.4 is used in many patients with SAH around the world, with several studies also attesting to this. Most of these studies are small sized (Moretti and Pizzi 2010; Lehmann et al. 2013; Anonymous 2012) (largest being 36 patients) and have not evaluated the effect of 6% HES 130/0.4 on complications associated with SAH like DCI, rebleeding, HCP, or death. This is of concern because recent large randomized control trials (RCTs) have shown that use of Voluven® and similar HES solutions have been associated with poor outcomes and increased mortality (Perner et al. 2011; Myburgh et al. 2012; Estrada and Murugan 2013) in patients with sepsis. Recently, the Surviving Sepsis campaign recommended against the use of HES in patients with severe sepsis (Dellinger et al. 2013). Although potato-derived and maize-derived HES have certain structural differences due to differences in the C2/C6 ratios and percentage of amylopectin content (Westphal et al. 2009), ex vivo studies have demonstrated no difference between the two with respect to bleeding (Godier et al. 2010). This may suggest a class effect by HES on outcomes like bleeding and acute kidney injury (AKI) (National Heart L and Blood Institute NHLBI and Food and Drug Administration FDA Public Workshop National Heart, Lung, and Blood Institute NHLBI and Food and Drug Administration FDA Public Workshop 2013).

The mechanism by which 6% HES 130/0.4 would increase SAH associated complications remains uncertain and under-researched. Only a handful of small studies (Hwang et al. 2007; Dieterich et al. 2003; Feng et al. 2010; Chen et al. 2009) have studied HES with respect to neurological pathophysiology. In a small study of 8 patients (Dieterich et al. 2003), it was found that higher molecular weight HES (Mw: 200 kDa) was not present in the CSF of patients who were given moderate amounts (500–1000 ml). The authors conceded in their study that the accumulation of HES in the brain interstitium could not be ruled out. Accumulation of HES in endothelial cells has been studied previously (Nohe et al. 2002), and it can be hypothesized that a similar accumulation of 6% HES 130/0.4 in the endothelium of the arachnoid villi might effect CSF absorption and promote hydrocephalus. Other mechanism by which HES could affect complications like DCI could be via inhibition of endothelium derived relaxation of blood vessels (Dagtekin et al. 2008; Dagtekin et al. 2010), which could have potentiated microcirculatory consequences.

We found that patients in our HES group with the primary composite outcome had a median rise in serum creatinine (∆Cr) of 12.5% during their hospital stay compared to a rise of 7.1% in the Non HES group. None of the patients required renal replacement therapy during their hospitalization. Although, the clinical significance of this rise is uncertain, retrospective data suggests that a decrease in creatinine clearance of as little as 25% can be associated with poor outcomes at 3 months following SAH (Zacharia et al. 2009). The increased risk of AKI with HES has been found previously by several studies (Estrada and Murugan 2013; Perner et al. 2012), and may be independent of molecular weight and substitution (National Heart L and Blood Institute NHLBI and Food and Drug Administration FDA Public Workshop National Heart, Lung, and Blood Institute NHLBI and Food and Drug Administration FDA Public Workshop 2013). It has been suggested that HES induced AKI may be due to HES accumulation in the proximal renal epithelial cells inducing an “osmotic nephrosis like lesion” leading to tubular obstruction and renal interstitial inflammation (Claus et al. 2010).

In our study, we found that patients in the HES group had more red blood cells transfused compared to the Non HES group. This result is similar to that of other studies (Myburgh et al. 2012; Estrada and Murugan 2013), where more blood product transfusions occurred in the 6% HES 130/0.4 group. HES has been found to be associated with platelet dysfunction, decrease in coagulation factors (like factor VIII and von Willebrand factor) and fibrin polymerization (Westphal et al. 2009; Godier et al. 2010). Although the effects on coagulation and bleeding are greater with higher molecular weight HES, even low molecular weight HES has been found to effect bleeding (Navickis et al. 2012). It is uncertain in our study whether increased red blood cell administration is a simple association with 6% HES 130/0.4 use or a result of it. It is well known that anemia commonly occurs after SAH in 39 to 57% patients (Giller et al. 1998; Kramer et al. 2008). Previous studies have found that lower levels of hemoglobin have been associated with poor outcomes in patients with SAH, including a higher rate of cerebral infarction (Naidech et al. 2006; Naidech et al. 2007; Kramer et al. 2009).This anemia is likely to reflect a suppression of bone marrow activity and response to erythropoietin (Corwin and Krantz 2000; Rodriguez et al. 2001) mediated by increased inflammatory cytokines such as interleukin-1 and tumor necrosis factor associated with SAH (Fink 2004; Gruber et al. 2000; Naredi et al. 2006). It can be further hypothesized that in patients with SAH, higher degree of anemia may represent a greater systemic inflammatory response (SIRS) and a greater likelihood of poor outcomes like cerebral ischemia and infarction (Naidech et al. 2006). Indeed 65.5% of the primary outcome (19 of 29 events) in our study occurred in patients which had a LHB of less than 9gdl^-1^. On analysis of the interaction between LHB and HES, we found an increased association between HES use and the primary outcome at higher levels of LHB. This relationship suggests that the effect of HES on the primary outcome is confounded by severity of SIRS at lower hemoglobin levels and unmasked only in patients where SIRS is less severe (as in patients with higher LHB levels).

Our study has several strengths. It is the first study that examines the safety of the use of 6% HES 130/0.4 (Voluven®) in patients with SAH. Secondly, we measure clinically important outcomes in our study, which are defined based on expert consensus and objective criteria (Naidech et al. 2005; Vergouwen et al. 2010). Thirdly, our data and outcomes represent real world practice because it is obtained from routinely collected clinical data.

Our study also has limitations. It is not a prospective trial. Therefore, the presence of unbalanced confounders and lack of randomization could have influenced the outcomes despite our efforts to measure and account for multiple important covariates during analysis. Nevertheless, data from our study will serve useful in designing a prospective RCT. Second, as in all database based studies errors in data collection and processing may exist. We tried to overcome these errors by using a quality controlled database and by performing additional data verifications after the initial data collection. Thirdly, pathophysiology explaining the results is unclear. Although there is supportive data for the hypothesis we offer as explanations, the exact biological mechanism can be a subject of further research. Lastly, we did not report serial monitoring of coagulation profiles during the hospital stay, although we did report blood product usage between the study groups. This is because our data was obtained from routine medical records and laboratory investigations.

In conclusion, our results suggest that caution should be exercised while utilizing 6% HES 130/0.4 in patients with SAH and a future adequately powered, prospective randomized clinical trial into the safety of 6% HES 130/0.4 in this patient population is warranted.

## Appendix 1: Definitions of outcomes

Delayed Cerebral Ischemia: Defined as the occurrence of focal neurological impairment or a decrease of at least 2 points on the Glasgow coma scale (either in total score or one of its individual components). This should have lasted at least 1 hour, was not apparent immediately after aneurysm occlusion, and could not be attributed to other causes (like bleeding or HCP) by means of clinical assessment, CT or MRI scanning of the brain, and appropriate laboratory studies.

Rebleeding: Defined as an acute deterioration in neurological status in conjunction with new hemorrhage apparent on CT while in the hospital.

HCP requiring CSF shunt: Recorded using appropriate Current Procedure Terminology (CPT) codes.

30 day mortality: Defined as a death occurring within 30 days of hospital admission. Outcome data was obtained from the DSR. The DSR contains updated death information for all patients which visited the Duke University Health System.

Peak Creatinine Rise (∆Cr): was calculated by the following formula:

$$\begin{array}{l} \mathrm{\Delta Cr}=(\mathrm{Peak}\;\mathrm{Serum}\;\mathrm{Creatinine}–\mathrm{Serum}\;\mathrm{Creatinine}\;\mathrm{at}\;\\ \;\mathrm{Admission})/\mathrm{Serum}\;\mathrm{Creatinine}\;\mathrm{at}\;\mathrm{Admission} \end{array}$$

## Authors’ original submitted files for images

Below are the links to the authors’ original submitted files for images. Authors’ original file for figure 1 Authors’ original file for figure 2
