# Supplementary figures and images for: Effect of 6% hydroxyethyl starch 130/0.4 in 0.9% sodium chloride (Voluven®) on complications after subarachnoid hemorrhage: a retrospective analysis
Source: Springerplus. 2013 Jul 15;2(1):314. doi: 10.1186/2193-1801-2-314 (PMC3717154; doi:10.1186/2193-1801-2-314)

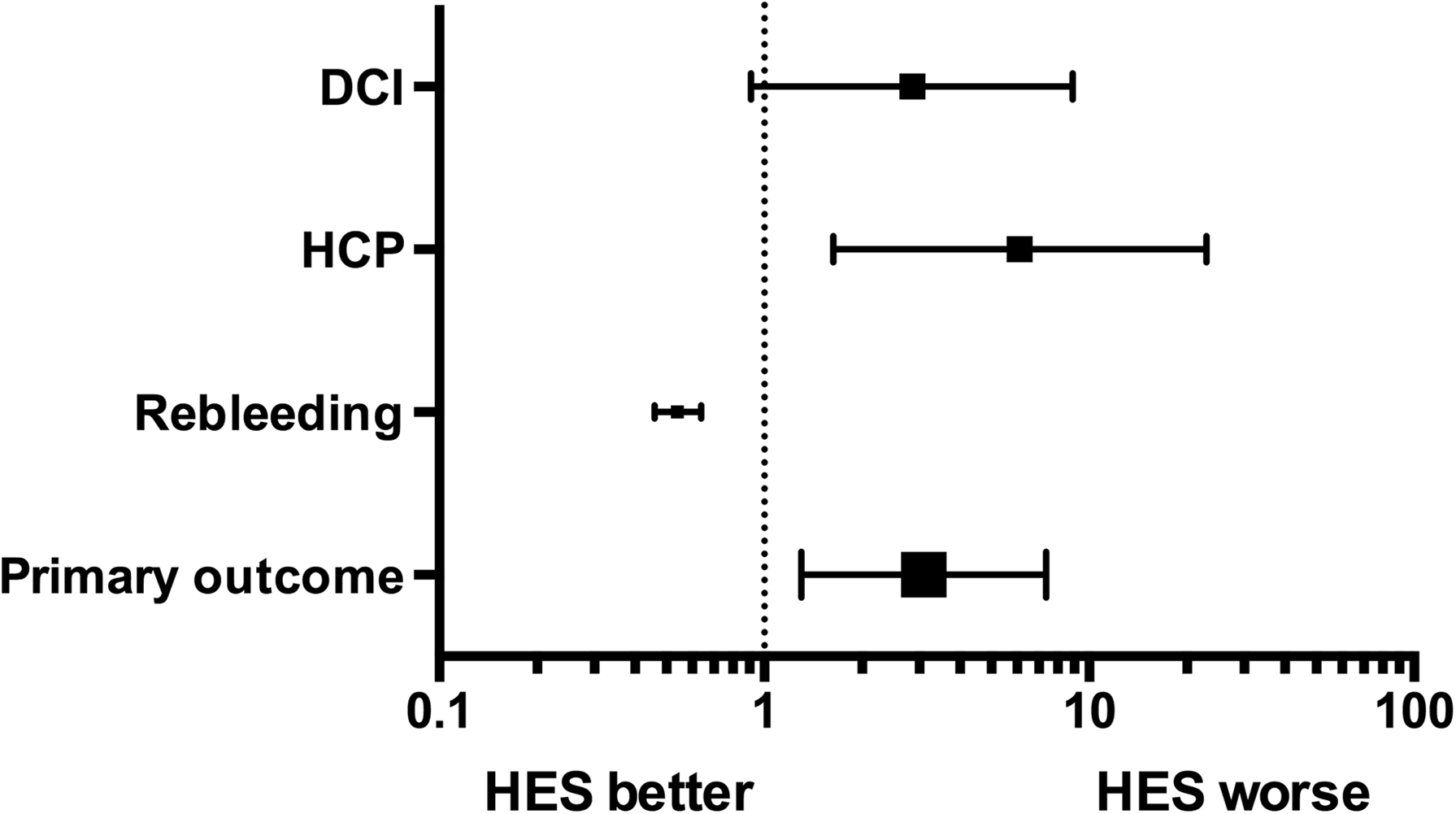

Supplement: Supplementary file 1 — Authors’ original file for figure 1 [file 40064_2013_392_MOESM1_ESM.tif]

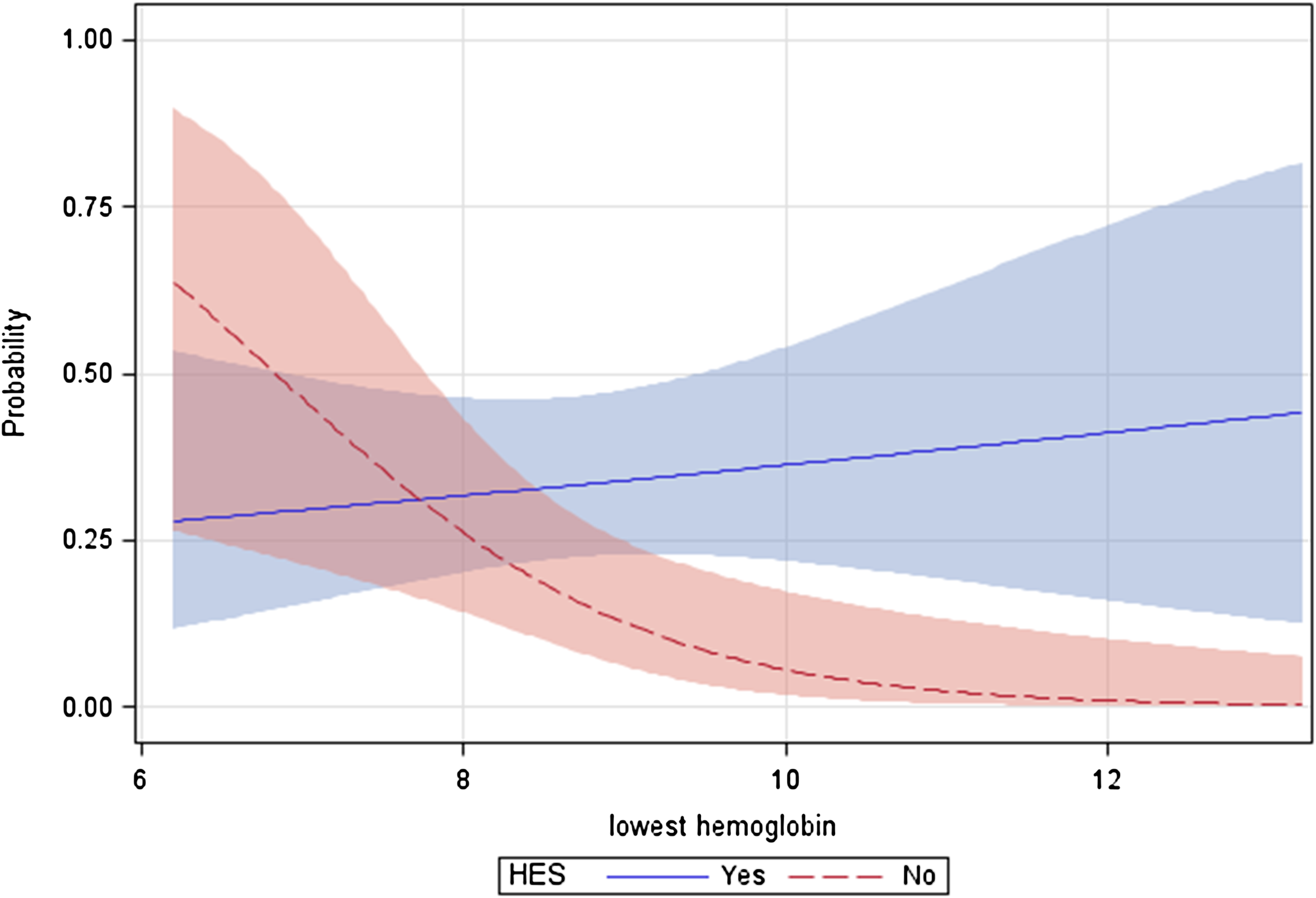

Supplement: Supplementary file 2 — Authors’ original file for figure 2 [file 40064_2013_392_MOESM2_ESM.tiff]
